# Effect of Soybean Oil on the Improvement of the Functionality of Edible Membrane-Type Food Packaging Films Based on Caseinate–Carboxymethyl Chitosan Compositions

**DOI:** 10.3390/membranes14050104

**Published:** 2024-04-30

**Authors:** Amal M. A. Mohamed, Hosahalli S. Ramaswamy

**Affiliations:** Department of Food Science and Agricultural Chemistry, Macdonald Campus of McGill University, 21111 Lakeshore Road, Ste Anne de Bellevue, QC H9X 3V9, Canada; amal.mohamed@mail.mcgill.ca

**Keywords:** caseinate, carboxymethyl, chitosan, soybean oil, water vapor permeability, mechanical properties, physical properties

## Abstract

Edible film biopolymers are gaining attention to tackle problems of plastic waste and food safety to alleviate environmental problems associated with plastic products in food packaging. In this study, caseinate–carboxymethyl chitosan (CA–CMCH) composite films were made with the incorporation of soybean oil (SO) using a casting technique. The influence of different soybean oil concentrations at 0, 0.5, and 1% (*w*/*w*) on physical, mechanical, barrier, and surface characteristics of films composed of caseinate–carboxymethyl chitosan (CA–CMCH) was evaluated. The brightest film (L* value of 95.95 ± 0.30) was obtained with the edible film made from the control group of samples with sodium caseinate (NaCA-100; 100% NaCA). The results also indicated that samples with 1% SO in NaCA-75 and CaCA-75 had lower water vapor permeability (WVP), while those with NaCA-50 and CaCA-50 showed higher values of WVP. For mechanical properties, this study found that incorporating soybean oil into the caseinate–carboxymethyl (CA–CMCH) composite films led to an enhancement of both tensile strength and elongation at break. The morphological structures, determined using SEM, of control and composite films showed compact and homogenous surfaces. Overall, the addition of soybean oil contributed to the improvement of the functional properties of the edible films, offering potential solutions to the environmental issues associated with plastic packaging and enhancing the safety and performance of food packaging.

## 1. Introduction

Food hydrocolloid polymers are promising ingredients used in different food formulations to replace petroleum-based polymers to reduce safety concerns. They offer an attractive alternative by including new biodegradable or renewable materials. The shift towards the use of bio-based food packaging materials has been increasing mainly due to the environmental concerns associated with plastic packaging materials and films [1]. Edible polymers also help to better control their transfer characteristics (e.g., moisture, gases, or flavor) across the packaging/films. Consequently, several studies have focused on the protective attributes of these barrier materials playing a key role in maintaining or improving food quality and extending the product shelf life [2]. Proteins, polysaccharides, and lipids are the major components of edible film materials [3]. Protein- and polysaccharide-based edible films have good mechanical and oxygen barrier characteristics; yet, they have a poor moisture barrier due to their inherent hydrophilic nature [4], while lipid-based edible films have favorable water vapor barrier properties but are more brittle [4].

Among the many biopolymers used, chitosan (CH) is an abundant polycationic biopolymer primarily derived from chitin in crustacean shells by the deacetylation of the amino groups. Chitosan and its derivatives are widely used in diverse biomedical applications due to their distinctive attributes such as biocompatibility, cohesion, non-toxicity, and capability to form gels. Additionally, they are good candidates for producing films, tablets, and nanotechnology-based systems, demonstrating potential for commercial production [5]. Recently, production and stabilization of emulsions by chitosan have been receiving increasing attention [6]. However, the majority of applications are limited due to their acidic pH, which limits their applications at higher pH.

Due to the rigid crystallinity of chitosan’s structure, it exhibits poor solubility in water, limiting its effectiveness in various processes. However, this problem has been addressed to create water-soluble chitosan [7]. The presence of hydroxyl and amino functional groups in chitosan’s structure leads to chemical modifications, which can enhance the soluble properties and electrical charge of chitosan [8]. Carboxymethylation as a hydrophilic substitution enhances the solubility and yield of carboxymethyl chitosan (CMCH), which has numerous biomedical applications [9,10].

Protein polymers have gained much attention in food packaging applications. Caseinates have been proposed as promising raw materials for the production of food packaging films due to the advantageous functional properties of proteins. These include the capacity to form networks, plasticity, elasticity, and effective barriers to oxygen, carbon dioxide, and aromas [11]. Hence, caseinate as an active material in the matrix could provide better barrier properties for modified atmosphere packaging. In addition, caseinates have been identified as effective environmentally friendly carriers of antimicrobial compounds [12]. The application of caseinates as edible and biodegradable films has been investigated, revealing good film-forming and coating abilities. These characteristics come from their open random coil structure and their ability to engage in intermolecular interactions, including hydrogen, electrostatic, and hydrophobic bonds. These types of interactions increase the inter-chain cohesion to make a film [13].

The incorporation of lipids into the hydrophilic film matrix is an interesting option to obtain films with enhanced moisture barrier characteristics. Lipids possess hydrophobic properties, which can contribute to the formation of a moisture barrier surface, but they are unable to form films on their own. Several lipids, including fatty acids, waxes, and vegetable oil, have been used as testing agents to reduce water vapor permeability in composite edible films from hydrophilic proteins. Soybeans are globally recognized as a major oil crop. Soybean oil (SO) is a commonly used main ingredient due to its functional properties. Soybean oil possesses significant amounts of unsaturated fatty acids, which contribute to its film-forming ability and flexibility. Also, soybean oil is particularly rich in unsaturated fatty acids, with about 85% mainly polyunsaturated fatty acids (PUFAs), like linolenic and linoleic acids, to meet nutritional needs. Moreover, it is high in triacyl-glycerol (constituting around 90–95%), phosphatides, tocopherols, and other fat-soluble compounds. The phosphatide content of SO, which mainly includes lecithin and cephalin, is approximately 2%. Overall, compared to other types of oils, soybean oil offers many advantages, providing many natural antioxidants like tocopherol [14]. Soybean oil exhibits good emulsifying properties, allowing it to form stable mixtures with other ingredients. Lipid composite edible films can be made either as emulsion or laminated films. Generally, laminated films demonstrate a significantly higher water vapor barrier ability compared to emulsion films [15].

The objective of this study was to first create stable emulsion films using caseinates (sodium and calcium caseinates) combined with carboxymethyl chitosan and then to evaluate the effect of adding the lipid component, i.e., soybean oil, to the matrix on the physical, mechanical, and water vapor permeability properties of the formed composite films. This is the first major study on CaCH/NaCH–CM–SO interactions, which are likely to play a major role in the film’s properties.

## 2. Materials

Carboxymethyl chitosan (DAC degree ≤ 85%) was obtained from Nutrakey Industries, Inc. (Qingdao, China). Glycerol, anhydrous calcium chloride, Tween 20, and sodium chloride were purchased from Sigma-Aldrich (Oakville, ON, Canada). Sodium caseinate (~92.0% protein) and calcium caseinate (~92.6% protein) were provided by CALDIC Canada Inc. (Mississauga, ON, Canada, http://www.caldic.com/en-ca). Soybean oil was obtained from Bulk Apothecary (Aurora, OH, USA).

## 3. Edible Film Formations

### 3.1. Preparation of Film-Forming Emulsions

Carboxymethyl chitosan (CMCH) edible film was prepared at a concentration of 2% *w*/*w* in water. The solution was stirred at 300 rpm for 4 h to dissolve the carboxymethyl chitosan. The solution was thereafter kept at 4 °C overnight. Caseinate edible films from sodium (NaCA) and calcium (CaCA) were prepared by dissolution in distilled water at 8% (*w*/*w*) for each one. Caseinate solutions were heated at 80 ± 2 °C for 30 min and then cooled to room temperature. Carboxymethyl chitosan and caseinate solutions were filtered through mesh 600 μm. Caseinates (CA) [NaCA or CaCA] and carboxymethyl chitosan (CMCH) solutions were mixed to provide a system with different caseinate chitosan combinations for both CaCA and NaCA: 100:0, 75:25, and 50:50 (*w*/*w*) Ca/CMCH. Briefly, 0.5% glycerol was added to all the mixtures and mixed for 15 min, and then Tween 20 was added to all solutions as an emulsifying agent. Further, these emulsions were homogenized with soybean oil (SO) at 0, 0.5, or 1% at 13,000 rpm using a tissue Tearor/Homogenizer (Biospec Products, INS. Model 985-370, Bartlesville, OK, USA) at room temperature for 15 min. The control film solutions were those with no (0%) soybean oil. Then, 14 mL of solutions were poured into polystyrene Petri dishes and dried at 45 °C for 8 h for casting. All films were stored at 25 °C and 50% RH for 48 h. Some of these methods have been reproduced from our previous publication [16].

### 3.2. Film Thickness

Sample film thickness was measured using a digital micrometer (Mitutoyo Manufacturing, Tokyo, Japan) with a sensitivity of 0.0001 mm. Measurements were made at least at ten random positions on each film. The average was used for the calculation of mechanical and water vapor permeability properties.

### 3.3. Film Solubility in Water

Film solubility (FS) was determined by the method of Leceta et al. [17], with some modifications. The film samples (1.5 cm × 4 cm) were weighed and then dried for 24 h at 105 °C; after that, the dried samples were immersed in 50 mL of distilled water. The tubes were covered and stirred at room temperature for 24 h. The undissolved parts of the film samples were dried at 110 °C for 24 h. Film solubility was calculated using the following equation:

(1) $$\%\;FS=\frac{w_{i}-w_{f}}{w_{i}}\times 100$$

where *w_i_* is the initial weight and *w_f_* is the weight of the undissolved film.

### 3.4. Moisture Content

The moisture content (MC) of the film samples was determined by weighing them initially (*w*_1_), followed by drying at 105 °C for 24 h and re-weighing (*w*_2_). Moisture content was measured for three replicates of each sample [18]. MC was calculated by using the following formula:

(2) $$\%\;MC=\frac{w_{i}-w_{f}}{w_{i}}\times 100$$

### 3.5. Color Measurement

The color parameters of the films were measured by a colorimeter (Minolta Crop., Ramsey, NJ, USA), and the CIELAB color parameters L*, a*, and b* were obtained. The total color difference (∆*E*) was calculated according to the following equation:

∆*E* = [(L_2_* − L_1_*)^2^ + (a_2_* − a_1_*)^2^ + (b_2_* − b_1_*)^2^]^1/2^ (3)

L_1_*, a_1_*, and b_1_* are the color parameters obtained from the standard white plate, and L_2_*, a_2_*, and b_2_* are the color parameters measured from the film samples [19].

### 3.6. Light Absorption

The light barrier characteristics of films were evaluated from their light absorption between wavelengths of 200 nm to 800 nm using a UV/Vis spectrophotometer (VWR, Model V-3100PC, Radnor, PA, USA) [18,20].

### 3.7. Water Vapor Permeability (WVP)

To measure the water vapor permeability (WVP) of the film samples, a gravimetric method was employed [19]. Glass cups were filled to one-fourth of the capacity with anhydrous calcium chloride to establish a relative humidity of 0% RH. The cups were covered with the test film samples, secured with a rubber band, and then placed in desiccators filled with saturated sodium chloride solution (75% RH). The covered cups were regularly weighed at different intervals (0, 1, 2, 4, 6, 9, and 12 h) at ambient temperature. Linear regression analysis was used to calculate the slope of weight loss over time. The WVP of the film samples was determined based on the weight gain of the cups, using the following equation:

WVP (g m^−1^ s^−1^ Pa^−1^) = WVTR × L/∆ P (4)

where WVTR is the water vapor transmission rate (g m^−2^ h^−1^), which is calculated from the slope of the regression line (g h^−1^) divided by the area (m^2^), L is the film thickness (m), and ∆ P is the water vapor pressure differential between the two sides of the film (Pa).

### 3.8. Mechanical Properties (TS, YM, and EAB)

The mechanical properties of the edible film samples (tensile strength (TS) and elongation at break (E)) were measured according to the ASTM standard method 638-10, with some modifications using an Instron Universal Testing Machine (Model 4500, Instron Corporation, Canton, MA, USA) at a crosshead rate of 50 mm/min. The tests were performed at room temperature.

### 3.9. Fourier-Transform Infrared Spectroscopy (FTIR)

FTIR spectra of the samples were evaluated by using an FTIR spectrometer (Agilent 5500a; Northern ANI Solutions, Santa Clara, CA, USA). The measurements were recorded between 4000 and 400 cm^−1^ at room temperature using OMNIC operating software (Version 7.3, Thermo Electron Corporation, Waltham, MA, USA), collected at a resolution of 4 cm^−1^ with 64 scans for each single run.

### 3.10. Scanning Electron Microscopy (SEM)

The microstructure surfaces and cross-sections of the dried edible films were determined using scanning electron microscopy (SEM) (TM3000, Hitachi High Technologies Corporation, Tokyo, Japan). The film samples were mounted on aluminum stubs using double-sided carbon adhesive tape.

### 3.11. Statistical Analysis

The SPSS software for MacBook version 29.0.0. (SPSS, Cary, NC, USA, 2023) was used to determine the effect of adding soybean oil to the edible film samples. Differences between treatments were tested for significance by a one-way ANOVA. Significant different means (*p* < 0.05) were separated by Duncan’s test.

## 4. Results and Discussion

### 4.1. Properties of CA/CMCH Composite Edible Films

#### 4.1.1. Film Thickness

Film thickness is a crucial physical property that significantly affects the rate of water vapor, gas, and other volatile compounds [21]. Table 1 shows the results for the film thickness as affected by process variables. The control sample (CMCH-100) and control sample incorporated with 0.05 and 1.0% SO (CMCH-0.05% and CMCH-1.0%) had lower thickness values (0.13 ± 0.09 mm), while the CA–CMCH samples with or without SO had much higher thickness (0.15–0.18 mm). In general, the addition of SO enhanced the film thickness for all samples. Within CMCH-100 samples, however, no significant differences in thickness were observed with the addition of soybean oil. There was also not much difference between NaCA and CaCA samples with different CMCH incorporations. This variation in thickness value may be due to the different densities of the solution used for film formation, emulsion status, air incorporation during the homogenization, etc., especially when oil is present. The film thickness formed in oil–water emulsions under a confined condition can be higher in particle size than the one formed in an aqueous condition [22]. Overall, the thickness of the film could be regulated by modifying the film ingredient concentrations during the formation of the film. The edible films may need a low water vapor permeability to ensure the preservation of product integrity and provide a package resistant to moisture. Conversely, good solubility is needed for films prior to use, especially when incorporating food additives [23].

#### 4.1.2. Film Solubility

All film samples were completely dissolved when prepared wet; so, the solubility results, also shown in Table 1, are based on samples after drying at 104 °C for 24 h. The solubility values ranged from 16.9% to 59.9%, indicating a significant (*p* < 0.05) variation in the cast edible film water solubility. The solubility of edible films made from caseinates and carboxymethyl chitosan can be an essential factor to consider when evaluating the film’s functionality and potential applications. Both caseinates and carboxymethyl chitosan are natural polymers derived from food sources and have unique properties that can contribute to the film’s solubility in water [6]. Our results indicated that the CMCH with 0.5% of SO had the highest solubility. However, with the addition of SO, the solubility decreased in proportion to the concentration of oil added. In general, edible films made from caseinates and carboxymethyl chitosan are expected to exhibit some level of water solubility, especially if a significant amount of carboxymethyl chitosan is used. This solubility feature presents advantages in specific applications, like food packaging, where the edible film-controlled dissolution can release bioactive compounds or enhance food preservation. However, from a stability point of view, lower water solubility would be desirable to prevent moisture pick up when the packages are stored under high relative humidity situations or when being used for products that are high in water activity. In this regard, both NaCA and CaCA with CMCH combinations and with or without incorporated oil provide a better film product.

#### 4.1.3. Moisture Content

Moisture content plays a critical role that contributes to the functionality of films. As shown in Table 1, the results indicate that the moisture content of the control sample (CMCH-100) was higher before than after adding oil to it. This can be expected because of the increased hydrophobicity of the sample with the addition of oil. Similar observations could also be made with different NaCA, CaCA, and CMCH combination films when SO was incorporated at 0.5 and 1.0% levels. The control samples had an average moisture content between 19.1 ± 0.80 and 38.5 ± 0.40%, whereas the oil-incorporated samples had an average moisture content between 5.96 ± 0.01 and 17.1 ± 0.55%. This suggests that oil incorporation into the matrix has a significant influence on the material’s ability to retain moisture. Within each sample category, however, the moisture content seemed to increase slightly with a higher concentration of soybean oil; however, this increase was not deemed statistically significant (*p* >0.05) in the case of CMCH-100 and at 75% for both NaCA and CaCA samples. Compared to the control sample, some data show that the incorporation of soybean oil into the biopolymers does not lead to an enhancement of the water-holding capability of the formed film, similar to the results observed by Song et al. [24], who carried out the work with wheat–corn starch films with lemon essential oil. In a different study [25], the treated samples with soybean oil showed an increase in the moisture content by increasing the oil concentration, and the authors reported that this could be attributed to the available bonding sites of CH, forming connections with water, leading to additional structural swelling. However, they had similar results when using rosemary oil with chitosan and sodium caseinate [25].

#### 4.1.4. Color

The color of edible films plays a key role in consumer preference for the acceptance of the products. Films with bright colors and glossy or transparent features are generally favored for better applications. Table 2 shows the data demonstrating the influence of using SO on the color characteristics of the (CA–CMCH) films.

The L* parameter results showed no significant differences (*p* > 0.05) in the treated film samples. However, the sodium caseinate (NaCA 100) and calcium caseinate (CaCA 100) edible films under control conditions had the highest L* value of 95.95 ± 0.30 and 95.74 ± 0.66, respectively. However, the statistical analysis using an ANOVA showed that the SO had an overall effect on the color results (*p* < 0.05) for caseinate samples. Giuseppe et al. [25] reported that chitosan with sodium caseinate films had similar results for the L value. The time it takes to dry and the temperature used for preparing drying of the film affect brightness parameters. The shorter the time and the lower the heat, the brighter the edible film.

The red to green color parameter a* ranged from −0.70 for the CMCH-100 film to −2.47 for the NaCA 100-0.5% film, while the yellow to blue color parameter b* was 11.59 for NaCA 100-0.5% and 1.89 for the CaCA100 edible film. A positive a* parameter means a red shade, while a negative a* value indicates a green shade. Similarly, a positive b* value means yellow and a negative b* value means a blue shade. The value of a* decreased negatively, and b* values increased on the positive side noticeably with additional soybean oil, providing green and yellow shades to the biopolymeric mixture films compared to the control values expected for CMCH 100. The absolute color difference (∆*E*) values, ranging from 2.50 to 12.04, showed a significant difference from the control. A similar behavior was observed in the WI results.

**Figure 1:**
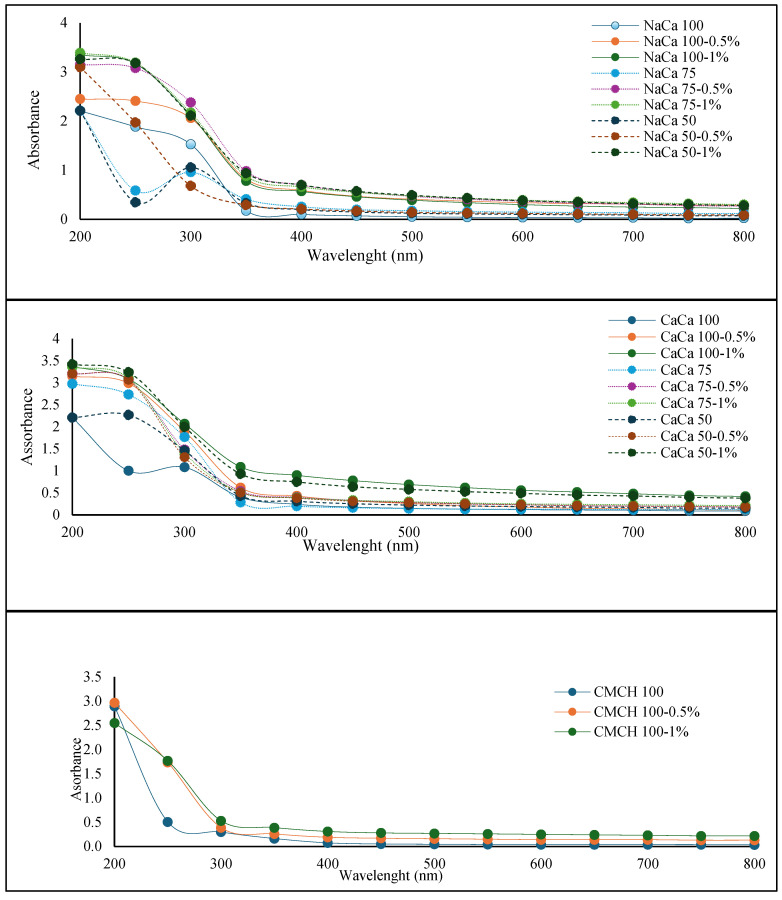
UV—Visible absorption spectra for caseinate–carboxymethyl chitosan (CA–CMCH) composite-based edible film incorporated with soybean oil at 0.5–1% SO.

The understanding and control of light absorption in edible films are crucial in various applications. For instance, in food packaging, the control of light absorption can enhance the shelf life of the packaged product by reducing degradation caused by light exposure. In addition, the edible film’s color and appearance contribute to the visual appeal of the food product, influencing consumer perception and acceptance. Figure 1 shows the UV–visible absorption spectra for (a) carboxymethyl chitosan-based films and (b) sodium caseinate- and calcium caseinate-based films with and without soybean oil at 0.5–1%. The data showed that the spectra of CMCH, NaCA, and CaCA at a ratio of 50 were similar. Other samples demonstrated a marked increase in the absorbance, and elevated absorbance levels were noticed for CaCA 100 and CaCA 75 for both their control and treated samples. All film spectra were high at a range of 200–350 nm. However, the visible absorption of each sample group showed no significant difference (*p* > 0.05).

### 4.2. Water Vapor Permeability (WVP)

**Figure 2:**
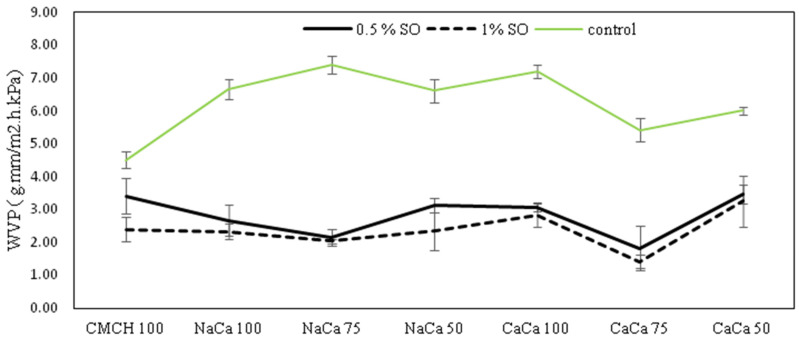
WVP of caseinate–carboxymethyl chitosan (CA–CMCH) composite edible films in the presence or absence of soybean oil at 0.5–1%.

Water vapor permeability (WVP) is critical to numerous industrial and research applications, especially in areas related to the packaging and preservation of various products. It plays a crucial role in maintaining the freshness, appearance, and overall integrity of packaged products over their intended storage or display period. The addition of soybean oil (SO) into the composite films of caseinates and carboxymethyl chitosan was intended to enhance the water vapor barrier property. This addition creates a hydrophobic barrier that leads to a reduction in water diffusion into the edible films. Figure 2 shows the WVP details of both control and emulsion edible films. The results show that the addition of SO significantly influenced the WVP (*p* < 0.05) of the prepared edible films. WVP properties are influenced by the nature of the biopolymers, which are primarily based on their moisture absorption capability at the film’s surfaces and the permeation capacity in film matrices [26]. Control films at 50% ratio for both sodium caseinate (NaCA 50) and calcium caseinate (CaCA 50) had greater WVP than other formed films, and a lower WVP was observed with treated samples at 75 ratios with 1% SO for both sodium caseinates (NaCA 75-1% SO) and calcium caseinates (CaCA 75-1% SO).

Using the SO in the film indicated a marked decrease in WVP, which is an enhancement of the water vapor barrier properties. Using soybean oil in edible film composites has a number of advantages. It makes the films stronger and better at keeping out moisture, which helps keep food fresh. On the other hand, using coconut oil in edible films has not yielded similar advantages. For example, adding coconut oil to soy protein isolate did not reduce the water vapor permeability (WVP) of the films compared to the control [27].

### 4.3. Mechanical Properties

**Figure 3:**
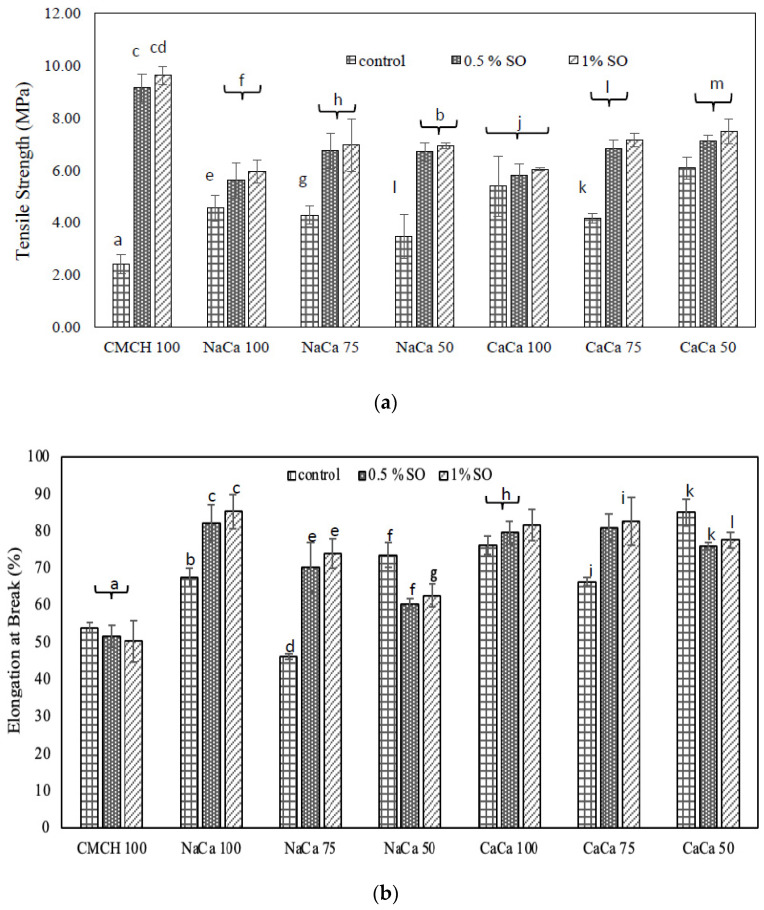
Mechanical properties: (**a**) tensile strength and (**b**) elongation at break of caseinate–carboxymethyl chitosan (CA–CMCH) composite-based edible films incorporated with soybean oil at 0.5–1% SO. Vertical bars represent standard error. Bars with different letters indicate significant differences at *p* < 0.05.

Mechanical properties such as the tensile strength of the film materials provide valuable information on the stretchability of the films. Other properties of materials like strength, ductility, and flexibility also can be determined by the mechanical spectra. The edible films need to withstand the various stresses during the transportation, storage, and handling of food. The mechanical characteristics of caseinate–carboxymethyl chitosan composite-based edible films incorporated with SO were evaluated as stress–strain curves, and the calculated elongation at break (εb) and tensile strength (TS) or stress at break (σb) are shown in Figure 3. The tensile strength and elongation at break of the formed edible films increased when SO was added. For tensile strength results, it can be seen that for the control samples of CMCH 100 and NaCA 100, both were significantly affected by using SO (*p* < 0.05), while CaCA 100 showed no effect. CA–CMCH composite films at 75% and 50% ratios and soybean oil at 0.5–1% had no significant influence on TS. Similar results were found for elongation at break (E) at 75%. A higher value of TS (9.6 MPa) was observed with CMCH 100-1% SO), while it was lower (2.4 MPa) for CMCH 100 (control). For elongation at break (εb), the values were 88% and 46.2% for NaCA 100-1% SO and NaCA 75 (control), respectively. However, a strong interaction will give the film a higher strength. These properties will influence their ultimate use as an effective edible film depending on the application. Moreover, many studies indicated that polymers like proteins, carbohydrates, and lipid combination matrices will increase their functionalities. The mechanical properties of materials are primarily influenced by both intermolecular and intramolecular distribution and density within the film network, which affect the flexibility and stretchability of the films.

### 4.4. FTIR

**Figure 4:**
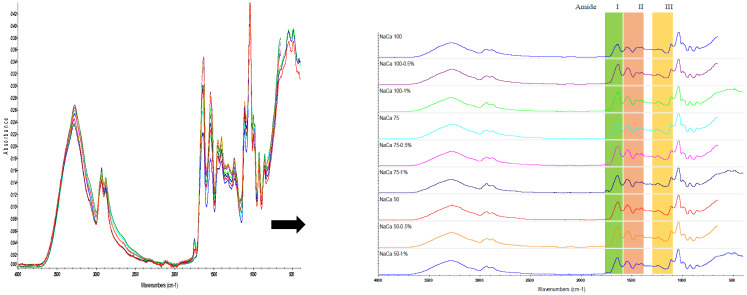

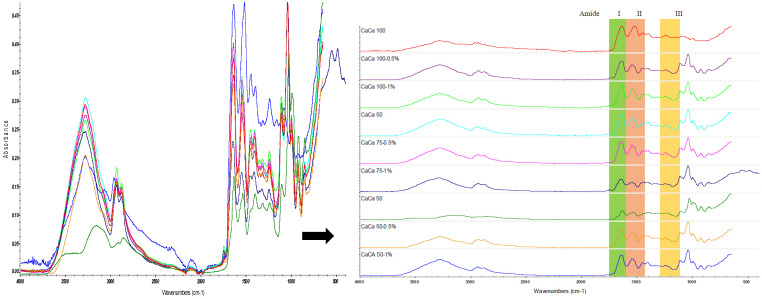

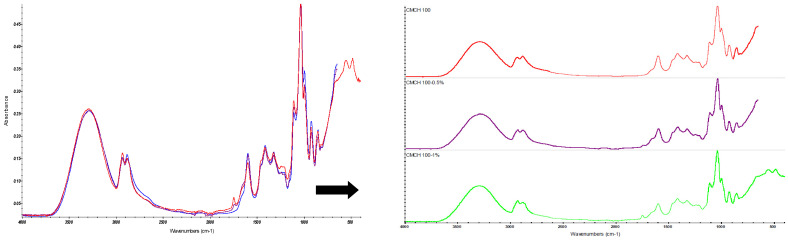
FTIR spectra of caseinate–carboxymethyl chitosan (CA–CMCH)-based edible film samples in the presence or absence of soybean oil at 0.5–1% SO.

**Figure 5:**
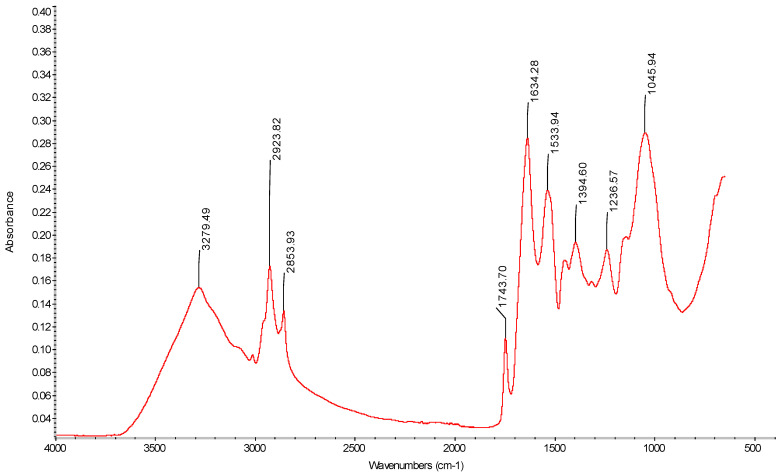
FTIR spectrum of fresh soybean oil (SO).

The FTIR spectra of edible films from caseinate–carboxymethyl chitosan (CA–CMCH) are shown in Figure 4, and Figure 5 shows the soybean oil FTIR spectrum. FTIR was used for the purpose of identifying the various chemical bonds and functional groups in the edible films, providing insights into their composition and potential interactions. The primary absorption peaks occurred within the spectral range of 800–1150 cm^−1^, indicating C-O-C stretching and C-C-C stretching vibrations, mostly associated with glycerol. The band at 1200–1250 cm^−1^ corresponds to the amide III band, arising from a combination of N-H and C-H vibrations. Amide I (1600–1700 cm^−1^) and amide II (1400–1550 cm^−1^) are attributed to C=O and C-N groups and N-H bending, respectively [28,29,30]. However, these characteristic bands are well-established in the analysis of protein-solvent interactions [31]. Both amide A (3842–3550 cm^−1^) and B (3000–2100 cm^−1^) reflect NH stretching vibrations [32,33,34].

**Figure 6:**
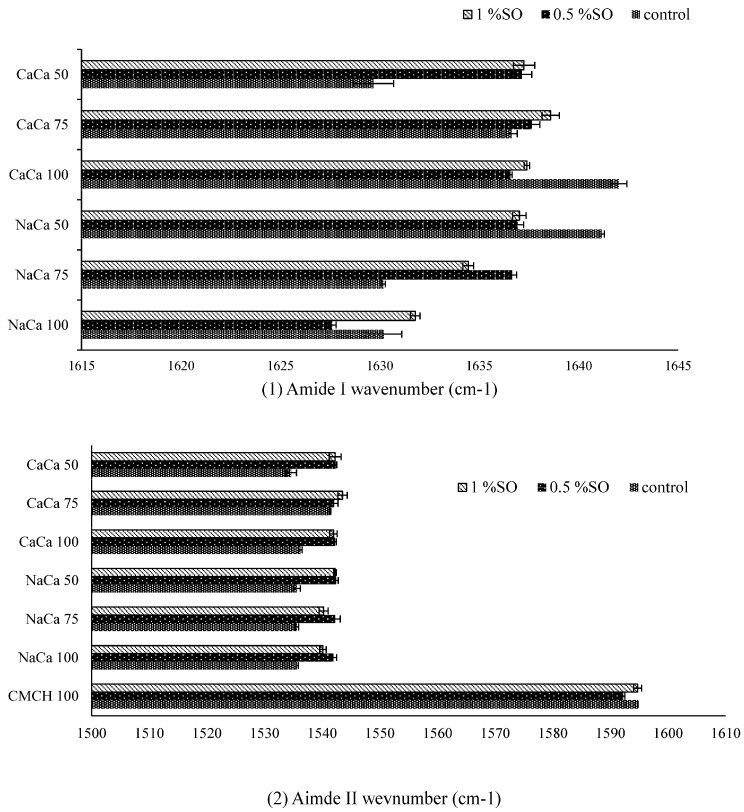
The amide I (**1**) and II peaks (**2**) of caseinate–carboxymethyl chitosan (CA–CMCH) control and treated samples with soybean oil at 0.5–1% SO.

Amide (I and II) bands are key components in the protein infrared spectrum, providing a strong correlation with the protein’s structure. In the spectral analysis of amide I and amide II, the peaks of the composite edible films changed in wavelength more than in the control films, which can be clearly seen in Figure 6. However, it was found that in all film samples, the intensity at the bands of amide A and amides I, II, and III increased at 0.5% SO and then decreased at 1% SO; for example, for the NaCA 100 sample, it increased from 0.25 g s^−1^ m^−2^ to 0.30 g s^−1^ m^−2^ and then decreased to 0.14 g s^−1^ m^−2^. However, no significant difference was observed in the intensity of peaks at 3273, 2929, and 1743 cm^−1^, which was related to soybean oil. Moreover, an increase or a decrease in the intensity of a peak in an FTIR spectrum implies that there is more or less absorption of infrared radiation at that specific wave number. The increase typically suggests that there is a higher concentration or greater presence of the functional group responsible for that absorption. For instance, if the peak corresponds to the C=O stretching vibration of a carbonyl group and its intensity increases, it could mean that there are more molecules with carbonyl groups in the sample, while the decrease in the intensity means fewer functional groups responsible for that absorption. If the peak belongs to O-H stretching vibrations of an alcohol group with lower intensity, it might indicate a decrease in the concentration of alcohol in the sample [32]. The FTIR analysis of the interaction between soybean oil and caseinate–carboxymethyl chitosan (CA–CMCH) has revealed shifts in absorption peaks and the emergence of new bands, indicating conformational changes and the potential formation of new complexes. These findings contribute to an understanding of how soybean oil and caseinate–carboxymethyl chitosan (CA–CMCH) interact at a molecular level and could have implications for the development of food products. In conclusion, the observed shifts in the FTIR spectra peaks and the emergence of new bands indicate conformational changes and potential complex formation. These findings deepen the understanding of molecular interactions between polymers, with implications for the development of food products.

### 4.5. Scanning Electron Microscopy

**Figure 7:**
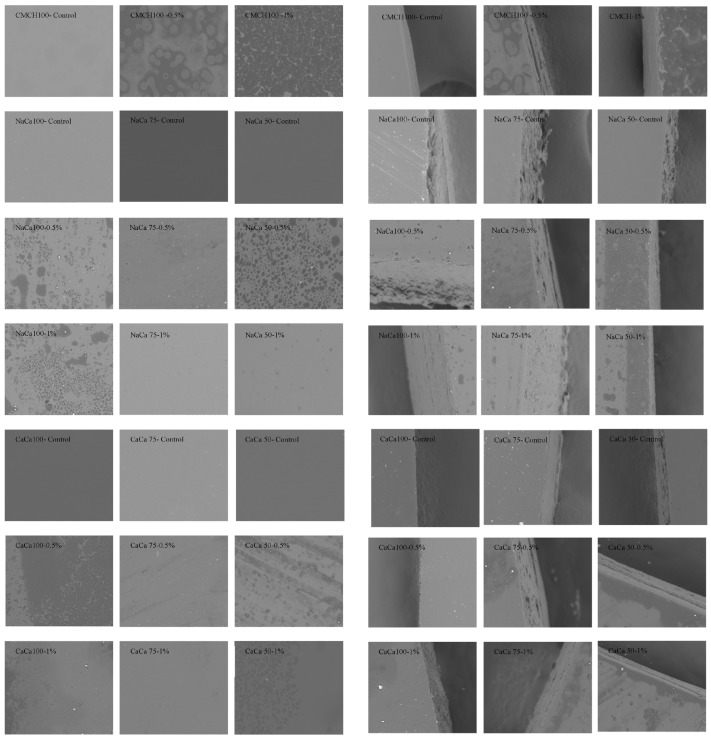
Scanning electron micrographs of caseinate–carboxymethyl chitosan (CA–CMCH) composite-based edible films with and without soybean oil at 0.5–1% SO: surfaces (magnification ×600) and cross-sections (magnification ×1200).

The SEM images for both surfaces and cross-sections of caseinate–carboxymethyl chitosan (CA–CMCH) composite-based edible films with soybean oil (SO) are shown in Figure 7. The microscopic views of the control samples CMCH 100, NaCA 100, and CaCA100 demonstrated a surface morphology that is smooth and uniform, with no apparent cracks, breaks, or hold on the surfaces, similar to edible film composites of NaCA and CaCA at 50 and 75 ratios. SEM surface images reveal a more compact and uniform film structure. The surface of the film appears smoother and more tightly knit, indicating better adhesion between the film components. However, the incorporation of soybean oil into the films showed that CMCH 100, NaCA 100, and CaCA 100 at two different concentrations of SO have smoother surfaces, with droplets of soybean oil on the surface. In addition, similar surfaces were observed with NaCA 50-0.5% and CaCA 50-0.5%. However, this could be related to the oils, which can migrate within the edible film over time due to factors such as temperature, drying rate, or ingredient interactions. This migration can cause the oil to move toward the film’s surface, resulting in the appearance of droplets. The results for other composite edible films revealed a smooth and uniform structure, devoid of any visual pores or cavities. This highlights a well-structured and compact film morphology when soybean oil is incorporated. The cross-sectional images of the edible film without oil were not tight and had some slight cracks. SEM analysis provides valuable insights into the surface characteristics of edible films, including the presence of an oily layer, emphasizing that SEM can aid in understanding the underlying causes of the issue and guide formulation and processing adjustments for improved product quality.

## 5. Conclusions

A composite edible film of caseinates (sodium caseinate and calcium caseinate), carboxymethyl chitosan, and SO was developed to enhance film properties, with a specific focus on barrier and mechanical attributes. The findings from this study highlighted the effectiveness of using soybean oil in the film solution, leading to improvements in the mechanical and barrier properties of composite edible films, which enhance their overall functionality. The observed changes in the physicochemical, mechanical, and barrier properties of caseinate–carboxymethyl chitosan composite films due to the inclusion of soybean oil indicate that the utilized concentrations are suitable for enhancing film transparency and tensile strength while improving water vapor barrier resistance. In this study, we illustrated the possibility of creating blended films from caseinate–carboxymethyl chitosan, integrated with soybean oil at low concentrations. These coating films may have promise for applications in food systems where films should dissolve during cooking or consumption. Furthermore, the composted films could be effective protective coatings for products inherently rich in lipids like nuts, cheeses, or meat. For edible packaging use, the results highlight the potential of the incorporation of soybean oil into different ratios of CA/CMCH composite films, leading to new materials with improved physicochemical properties, such as mechanical and barrier properties. Furthermore, these composite films may be used for the development of applications in food packaging with the aim of extending the shelf life of food products.

## Figures and Tables

**Table 1:** Film thickness, water solubility, and moisture content of film samples incorporated with 0.5–1% soybean oil. The numbers 100, 75, and 50 refer to the proportion of NaCA or CaCA in the formulation.

Sample	Thickness (mm)	Solubility in Water (%)	Moisture Content (%)
CMCH 100	0.13 ± 0.09 ^a^	55.93 ± 0.33 ^a^	22.58 ± 0.19 ^a^
CMCH 100-0.5%	0.13 ± 0.02 ^a^	59.91 ± 0.8 ^b^	5.96 ± 0.01 ^b^
CMCH 100-1%	0.13 ± 0.10 ^a^	47.16 ± 0.09 ^c^	6.78 ± 0.04 ^bn^
NaCA 100	0.16 ± 0.07 ^b^	39.04 ± 0.40 ^d^	38.50 ± 0.40 ^c^
NaCA 100-0.5%	0.16 ± 0.03 ^b^	16.94 ± 0.39 ^e^	11.05 ± 0.2 ^d^
NaCA 100-1%	0.17 ± 0.47 ^c^	22.62 ± 0.83 ^f^	15.53 ± 0.05 ^e^
NaCA 75	0.16 ± 0.08 ^b^	33.84 ± 0.51 ^g^	24.19 ± 0.26 ^f^
NaCA 75-0.5%	0.16 ± 0.03 ^b^	27.56 ± 0.1 ^h^	11.67 ± 0.01 ^g^
NaCA 75-1%	0.17 ± 0.04 ^c^	27.11 ± 0.87 ^h^	10.69 ± 0.05 ^g^
NaCA 50	0.15 ± 0.21 ^d^	37.60 ± 2.00 ^i^	21.32 ± 0.27 ^h^
NaCa 50-0.5%	0.17 ± 0.22 ^c^	40.32 ± 0.02 ^j^	10.03 ± 0.07 ^io^
NaCA 50-1%	0.18 ± 0.01 ^e^	30.81 ± 0.6 ^k^	13.38 ± 0.05 ^j^
CaCA 100	0.16 ± 0.01 ^b^	36.48 ± 0.35 ^i^	33.37 ± 0.23 ^k^
CaCA 100-0.5%	0.17 ± 0.03 ^c^	30.23 ± 0.65 ^k^	10.40 ± 0.05 ^e^
CaCA 100-1%	0.18 ± 0.08 ^e^	29.57 ± 0.01 ^k^	17.14 ± 0.55 ^l^
CaCA 75	0.16 ± 0.05 ^b^	33.39 ± 0.31 ^g^	22.98 ± 0.25 ^f^
CaCA 75-0.5%	0.16 ± 0.01 ^b^	31.64 ± 0.02 ^k^	8.71 ± 0.05 ^m^
CaCA 75-1%	0.17 ± 0.06 ^c^	28.90 ± 0.34 ^l^	9.73 ± 0.11 ^m^
CaCA 50	0.15 ± 0.01 ^d^	30.34 ± 0.14 ^k^	19.07 ± 0.80 ^n^
CaCA 50-0.5%	1.6 ± 0.03 ^b^	37.43 ± 0.9 ^i^	7.82 ± 0.03 ^b^
CaCA 50-1%	0.16 ± 0.9 ^b^	30.25 ± 0.76 ^k^	10.03 ± 0.06 ^o^

Mean values and (standard deviation). Different superscripts within the same column indicate statistically significant differences at the level of 5% significance.

**Table 2:** Color parameter, total color, and white index difference of edible film samples incorporated with 0.5–1% soybean oil. The numbers 100, 75, and 50 refer to the proportion of NaCA or CaCA in the formulation.

Sample	L*	a*	b*	∆E	WI
CMCH 100	91.1 ± 0.16 ^af^	−0.70 ± 0.2 ^fhi^	7.39 ± 0.06 ^a^	9.41 ± 0.05 ^ag^	88.7 ± 0.81 ^ah^
CMCH 100-0.5%	90.7 ± 0.37 ^ab^	−0.71 ± 0.05 ^ehi^	6.20 ± 0.51 ^bj^	5.98 ± 0.31 ^e^	88.8 ± 0.34 ^abc^
CMCH 100-1%	91.3 ± 0.15 ^abcd^	−0.91 ± 0.07 ^cg^	6.88 ± 0.73 ^ab^	8.83 ± 0.66 ^ab^	88.9 ± 0.55 ^abc^
NaCA 100	96.0 ± 0.30 ^ce^	−0.43 ± 0.16 ^bi^	2.11 ± 0.15 ^f^	2.50 ± 0.11 ^f^	96.4 ± 0.11 ^j^
NaCA 100-0.5%	89.2 ± 0.57 ^df^	−2.47 ± 0.13 ^df^	11.6 ± 0.64 ^e^	8.86 ± 0.33 ^ab^	83.9 ± 0.53 ^e^
NaCA 100-1%	91.1 ± 1.02 ^abcd^	−1.82 ± 0.05 ^abcd^	8.6 ± 0.29 ^c^	10.44 ± 0.57 ^h^	87.5 ± 0.73 ^gh^
NaCA 75	90.2 ± 0.47 ^ab^	−0.93 ± 0.04 ^gh^	5.5 ± 0.51 ^ij^	8.65 ± 0.10 ^abc^	89.2 ± 0.3 ^abc^
NaCA 75-0.5%	91.0 ± 0.43 ^abc^	−2.22 ± 0.21 ^efh^	10.6 ± 0.77 ^d^	7.76 ± 0.58 ^cd^	85.96 ± 0.89 ^f^
NaCA 75-1%	91.2 ± 1.40 ^abcd^	−2.03 ± 0.05 ^cei^	10.5 ± 0.11 ^d^	12.0 ± 0.63 ^i^	86.2 ± 0.88 ^f^
NaCA 50	90.2 ± 0.22 ^abcd^	−1.17 ± 0.11 ^g^	3.9 ± 0.18 ^h^	6.55 ± 0.30 ^d^	88.0 ± 0.48 ^d^
NaCA 50-0.5%	91.5 ± 0.86 ^abcd^	−1.89 ± 0.01 ^bc^	10.0 ± 0.15 ^d^	7.35 ± 0.11 ^d^	86.7 ± 0.59 ^fg^
NaCA 50-1%	91.5 ± 0.87 ^abcd^	−1.90 ± 0.02 ^bc^	10.0 ± 0.16 ^d^	11.5 ± 0.43 ^i^	86.7 ± 0.59 ^fg^
CaCA 100	95.7 ± 0.66 ^e^	−0.82 ± 0.08 ^hi^	1.9 ± 0.25 ^fg^	3.12 ± 0.18 ^f^	94.6 ± 0.62 ^j^
CaCA 100-0.5%	91.4 ± 0.49 ^abcd^	−1.63 ± 0.13 ^abd^	5.3 ± 0.58 ^i^	7.73 ± 0.72 ^cd^	89.7 ± 0.69 ^bd^
CaCA100-1%	90.8 ± 1.30 ^abc^	−1.47 ± 0.15 ^a^	6.5 ± 0.30 ^ab^	9.01 ± 0.56 ^abg^	88.6 ± 0.84 ^ab^
CaCA 75	90.8 ± 0.31 ^a^	−1.35 ± 0.13 ^a^	4.8 ± 0.53 ^hi^	7.54 ± 0.48 ^bc^	88.17 ± 0.33 ^abcd^
CaCA 75-0.5%	92.0 ±0.16 ^bcd^	−1.50 ± 0.08 ^a^	7.0 ± 0.08 ^ab^	8.60 ± 0.09 ^ac^	89.26 ± 0.10 ^abcd^
CaCA 75-1%	92.0 ± 0.13 ^bcd^	−1.51 ± 0.02 ^a^	7.0 ± 0.33 ^ab^	8.74 ± 0.34 ^ab^	89.11 ± 0.31 ^abcd^
CaCA 50	91.7 ± 0.20 ^bcd^	−0.91 ± 0.05 ^g^	4.0 ± 0.09 ^g^	5.09 ± 0.10 ^e^	90.07 ± 0.11 ^i^
CaCA 50-0.5%	92.4 ± 0.30 ^d^	−1.47 ± 0.03 ^a^	7.2 ± 0.21 ^a^	8.52 ± 0.27 ^bc^	89.47 ± 0.29 ^abcd^
CaCA 50-1%	92.1 ± 0.36 ^cd^	−1.58 ± 0.02 ^ad^	8.4 ± 0.35 ^c^	9.75 ± 0.38 ^gh^	88.37 ± 0.38 ^abh^

Letters in the same column sharing the same alphabet are not significantly different from each other (*p* > 0.05).

## Data Availability

The original contributions presented in the study are included in the article, further inquiries can be directed to the corresponding author.
